# Bohring-Opitz syndrome caused by a novel ASXL1 mutation (c.3762delT) in an IVF baby: A case report

**DOI:** 10.1097/MD.0000000000028759

**Published:** 2022-02-04

**Authors:** Dongbo Wang, Xin Yuan, Haichun Guo, Shuyuan Yan, Guohong Wang, Yanling Wang, Tuanmei Wang, Jun He, Xiangwen Peng

**Affiliations:** a Changsha Hospital for Maternal and Child Health Care of Hunan Normal University, China; b The First Hospital of Hunan University of Chinese Medicine, China.

**Keywords:** ASXL1, IVF, whole-exome sequencing

## Abstract

**Rationale:**

Bohring-Opitz syndrome is a severe congenital disorder associated with a de novo mutation in the additional sex combs-like 1 (ASXL1) gene, and it is characterized by symptoms that include developmental delay and musculoskeletal and neurological features.

**Patient concerns:**

The patient was a girl, an in vitro fertilization (IVF) baby, with delayed motor development, drooling, short stature, slow growth, low muscle tone, image diagnosis of hypoplasia of the corpus callosum, delayed tooth eruption, high palatal arch, adduction of the thumb, drooling, not chewing, excessive joint activity, and ligament relaxation.

**Diagnosis:**

Whole-exome sequencing analysis detected 1 novel disruptive frameshift mutation in ASXL1 in the proband but wild-type ASXL1 in both parents.

**Interventions:**

Approximately 1 year of rehabilitation training, which included exercise therapy, toy imitation operation, cognition of daily objects, daily living skills training, gesture language training, oral muscle training, and hand movement training.

**Outcomes:**

After approximately 1 year of training, the patient was 3 years old and able to eat normally without drooling. She was able to grasp objects and pick them up after they fell. She was able to grasp small objects and actively played with toys. In addition, she was able to crawl on the floor (at slow speed, with poor initiative), stand with assistance, and walk with assistance; she was unstable when standing unassisted (standing unassisted for 8 seconds at most during training).

**Lesson:**

ASXL1 c.3762delT is a novel mutation that may be caused by IVF. This finding suggests that appropriate gene mutation detection approaches may be necessary for IVF technology.

## 1 Introduction

Bohring-Opitz syndrome (BOS) was first described by Bohring et al in 1999.^[1–4]^ The patients they described had several features in common, including a prominent metopic suture, hypertelorism, exophthalmos, cleft lip and palate, limb anomalies, difficulty feeding, and severe developmental delays.^[5]^ In almost 50% of cases that meet the clinical criteria for BOS, de novo frameshift and nonsense mutations in the additional sex combs-like 1 (ASXL1) gene have been detected,^[2,6–14]^ suggesting that loss of function of this gene is the major cause. We report the clinical characterization of a small baby who had total growth retardation, agenesis of the corpus callosum, and hypotonia and was clinically diagnosed with BOS. Whole-exome sequencing analysis revealed 1 novel disruptive frameshift deletion in the ASXL1 gene. The child was conceived through in vitro fertilization (IVF), and both parents are wild-type for ASXL1. Genetic risks and pre-implantation genetic testing in IVF have been the focus of many studies.^[15–17]^ In our study, a novel disruptive frameshift of ASXL1 was found in an IVF baby, which may have been caused by IVF and suggests that appropriate pre-implantation genetic testing is necessary before embryo transfer.

## 2 Case report

The proband was an 11-month-old girl, born 33 weeks premature and conceived through IVF. She exhibited the following features: delayed motor development, drooling, short stature, slow growth, low muscle tone, hypoplasia of the corpus callosum as determined by image diagnosis, delayed tooth eruption, high palatal arch, adduction of the thumb, excessive joint activity, and ligament relaxation.

**Figure 1:**
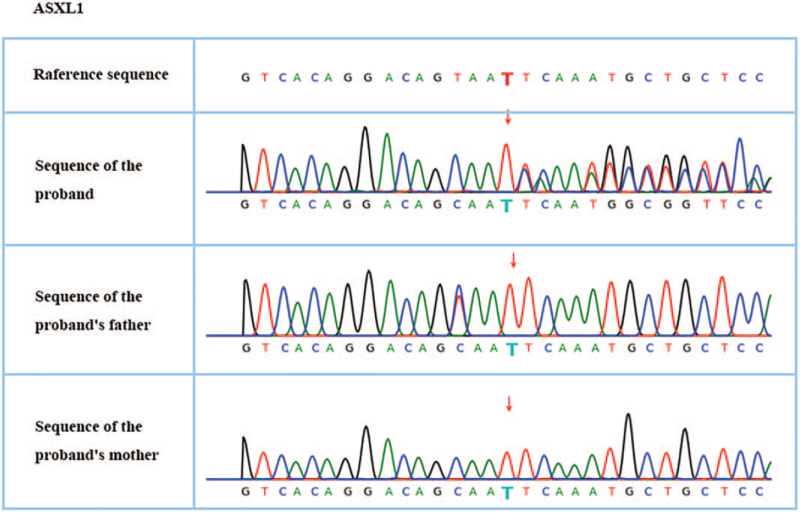
First-generation sequencing verifies a disease-causing mutation in the proband and family members: c.3762delT (p.N1254fs).

Whole-exome sequencing and whole-genome copy number variation detection were performed. Written informed consent was obtained from the patient's family. A de novo frameshift deletion of ASXL1, c.3762delT (p. N1254fs), was identified (Fig. 1). According to the American Academy of Medical Genetics and Genomics guidelines,^[18]^ the c.3762delT mutation of the ASXL1 gene is suggested as a pathogenic site related to the autosomal dominant genetic disease BOS. The clinical features consistent with the phenotype of this case are overall developmental delay, corpus callosum hypoplasia, and hypotonia. We did not detect chromosomal aneuploidy or genome copy number variants of 100 kb or more that are known to cause disease. The first-generation sequencing results showed that the proband had c.3762delT heterozygosity in the ASXL1 gene, and neither the father nor the mother was a carrier (Fig. 1).

### 2.1 Copy number variant results

**Figure 2:**
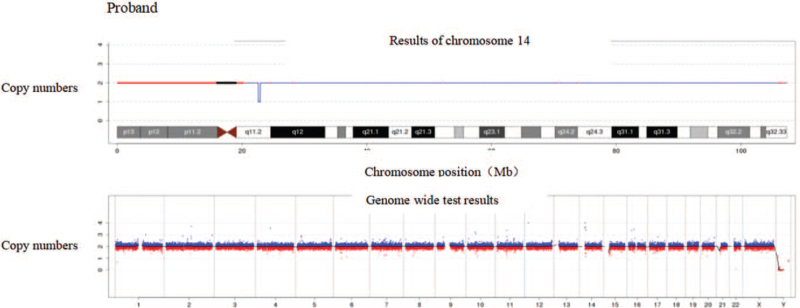
CNV-seq results of the proband: CNV-seq results showed that the proband had a chromosomal deletion SEQ [HG19] Del (14) (Q11.2) CHR14: G.22620001_22940000Del, but there is no known gene in this region. CNV = copy number variant.

The 0.32 Mb region at q11.2 on chromosome 14 was missing. Querying of the DGV, DECIPHER, OMIM, UCSC, and PubMed public database resources revealed that this fragment does not contain any known genes (Fig. 2).

When she was 2 years old, her wrists and knuckles were mostly in flexion, her initiative was very low, her hand movement was poor, she could not grasp items, her holding time was not long, her percussion strength was weak, and she could not crawl or walk. Regarding oral features, she exhibited a high, narrow jaw arch, low sensory sensitivity, and no chewing.

Interventions: At the age of 2 years, rehabilitation training was conducted in the children's rehabilitation department and included exercise therapy, toy imitation operation, cognition of daily objects, daily living skills training, gesture language training, oral muscle training, and hand movement training.

Outcomes: After approximately 1 year of training, at the age of 3 years old, she was able to eat normally without drooling. She was able to grasp objects and pick them up after they fell. She was able to grasp small objects and actively played with toys. In addition, she was able to crawl on the floor (at slow speed, with poor initiative), stand with assistance, and walk with assistance; she was unstable when standing unassisted (standing unassisted for 8 seconds at most during training).

## 3 Materials and methods

### 3.1 IVF procedures

The protocol for a modified natural cycle IVF was approved by the institutional review board of the Reproductive and Genetic Hospital of CITIC-Xiangya. Modified natural IVF cycles were conducted as follows: natural ovulatory cycles were monitored with serial transvaginal ultrasound examinations and serum E2 determinations. When the lead follicle reached pre-ovulatory status according to the cycle day, ultrasound, and E2 levels,^[19,20]^ 0.25 mg of the GnRH antagonist ganirelix acetate was administered along with 200 IU of human chorionic gonadotropin. Both medications were self-administered subcutaneously by the prospective. The prospective parents were asked to return daily for continued serial monitoring with ultrasound and serum E2. Both medications were administered daily until follicle maturity criteria were reached. These criteria were previously established by our program as a guideline for the timing of ovulation triggering and comprised combinations of follicle size and E2 level (>20 mm and >200 pg/mL, >18 mm and >250 pg/mL, or >15 mm and >300 pg/mL for mean follicle diameter and serum E2 level, respectively). When these maturity criteria were reached, a trigger dose of 10,000 IU of human chorionic gonadotropin was administered. Follicle aspiration, fertilization in vitro, and embryo transfer were subsequently performed as previously described.^[20,21]^

### 3.2 Ethical review

The Ethics Committee of Changsha Maternal and Child Health Hospital approved this study.

## 4 Discussion

Is IVF completely safe? This is a controversial issue. Some studies have shown that IVF is safe,^[22,23]^ whereas others have indicated that various assisted reproductive technologies (ARTs) can induce local and functional epigenetic abnormalities, especially DNA methylation and H3K4me3, providing an epigenetic basis for potential long-term health risks in ART-conceived offspring.^[24]^ We found a novel mutated gene in an IVF baby, which indicated that ARTs may cause mutations; however, a larger sample is needed to draw conclusions.

Some of the clinical features of our proband were consistent with previous BOS findings, including full-scale developmental delay and agenesis of the corpus callosum.^[4]^ Furthermore, exon sequencing of the whole gene showed that the ASXL1 gene had a c.3762delT frameshift mutation. However, the head sizes of our proband were normal, which is contrary to the microcephaly reported in every other case with ASXL1 disease-causing variants.^[25,26]^

ASXL1 encodes a chromatin-binding protein required for normal determination of segment identity in the developing embryo.^[27]^ The protein is a member of the polycomb group of proteins, which are involved in embryogenesis and carcinogenesis through transcriptional regulation of target genes.^[28]^ The ASXL1 protein is thought to disrupt chromatin in localized areas, enhancing the transcription of certain genes while repressing the transcription of others.^[29]^ The protein encoded by ASXL1 functions as a ligand-dependent coactivator for retinoic acid receptors in cooperation with nuclear receptor coactivators.^[30,31]^

The mutation of ASXL1 in our case (c.3762delT) was identified as a new mutation in the public human exon database. The deletion occurred in exon 12 and caused changes in the reading frame, leading to changes in protein function. Similar to all known frame-coding mutations, most frame-coding mutations in ASXL1 alter protein function, and most such mutations cause BOS. The frameshift mutations c.3754_3758del (p.gln1251_asp1252inster) and c.3769del (p.aLA1257fs) occur in the last exon of ASXL1, and both lead to BOS as revealed by ClinVar. These phenotypes are similar to the mutation we have found. This observation suggests that mutations in the latter part of the gene can lead to BOS.

We conclude that the c.3762delT mutation is a disease-causing mutation responsible for the patient's BOS clinical presentation. The molecular diagnostic methods currently available have not only paved the way for accurate diagnoses and genotype–phenotype correlations but also helped delineate locus heterogeneity among highly similar phenotypes. Hence, accurate phenotypic characterization is essential for choosing the appropriate molecular diagnostic method.

## Acknowledgments

We thank all of the patients and healthy volunteers who agreed to participate in the present study and all those who helped us successfully complete the research.

## Author contributions

**Conceptualization:** Xin Yuan, Tuanmei Wang, Dongbo Wang.

**Data curation:** Jun He, Dongbo Wang, Shuyuan Yan, Guohong Wang.

**Formal analysis:** Xin Yuan, Tuanmei Wang, Dongbo Wang.

**Funding acquisition:** Tuanmei Wang.

**Investigation:** Dongbo Wang, Haichun Guo.

**Methodology:** Yanling Wang.

**Writing – review & editing:** Xiangwen Peng.

## References

[1] Hastings R Cobben JM Gillessen-Kaesbach G. (Oberklaid-Danks) syndrome: clinical study, review of the literature, and discussion of possible pathogenesis. Eur J Hum Genet 2011;19:513–9. 21368916 10.1038/ejhg.2010.234 PMC3083618

[2] Prats-Martin C Burillo-Sanz S Morales-Camacho RM. ASXL1 mutation as a surrogate marker in acute myeloid leukemia with myelodysplasia-related changes and normal karyotype. Cancer Med 2020;9:3637–46. 32216059 10.1002/cam4.2947 PMC7286456

[3] Inoue D Matsumoto M Nagase R. Truncation mutants of ASXL1 observed in myeloid malignancies are expressed at detectable protein levels. Exp Hematol 2016;44:172–6.e1. 26700326 10.1016/j.exphem.2015.11.011

[4] Dangiolo SB Wilson A Jobanputra V Anyane-Yeboa K. Bohring-Opitz syndrome (BOS) with a new ASXL1 pathogenic variant: review of the most prevalent molecular and phenotypic features of the syndrome. Am J Med Genet A 2015;167A:3161–6. 26364555 10.1002/ajmg.a.37342

[5] Bohring A Oudesluijs GG Grange DK Zampino G Thierry P. New cases of Bohring-Opitz syndrome, update, and critical review of the literature. Am J Med Genet A 2006;140:1257–63. 16691589 10.1002/ajmg.a.31265

[6] Richards S Aziz N Bale S. Standards and guidelines for the interpretation of sequence variants: a joint consensus recommendation of the American College of Medical Genetics and Genomics and the Association for Molecular Pathology. Genet Med 2015;17:405–24. 25741868 10.1038/gim.2015.30 PMC4544753

[7] Abdel-Wahab O Adli M LaFave LM. ASXL1 mutations promote myeloid transformation through loss of PRC2-mediated gene repression. Cancer Cell 2012;22:180–93. 22897849 10.1016/j.ccr.2012.06.032 PMC3422511

[8] Yang H Kurtenbach S Guo Y. Gain of function of ASXL1 truncating protein in the pathogenesis of myeloid malignancies. Blood 2018;131:328–41. 29113963 10.1182/blood-2017-06-789669 PMC5774208

[9] Uni M Masamoto Y Sato T. Modeling ASXL1 mutation revealed impaired hematopoiesis caused by derepression of p16Ink4a through aberrant PRC1-mediated histone modification. Leukemia 2019;33:191–204. 29967380 10.1038/s41375-018-0198-6

[10] Takeda R Asada S Park SJ. HHEX promotes myeloid transformation in cooperation with mutant ASXL1. Blood 2020;136:1670–84. 32492700 10.1182/blood.2019004613

[11] Montes-Moreno S Routbort MJ Lohman EJ. Clinical molecular testing for ASXL1 c.1934dupG p. Gly646fs mutation in hematologic neoplasms in the NGS era. PLoS One 2018;13:e0204218. 30222780 10.1371/journal.pone.0204218 PMC6141087

[12] Fang L Cui Y Mi Y. Somatic ASXL1 p.R693X mutation identified by next generation sequencing in isolated myeloid sarcoma involving the mediastinum. Curr Med Res Opin 2020;36:1003–7. 32286099 10.1080/03007995.2020.1744121

[13] Elevated blood sugar levels lower brain function in diabetics. The effects of diet on your blood glucose levels can diminish memory and other cognitive skills. Duke Med Health News 2009;15:09–10. 19579316

[14] Zhao W Hu X Liu Y. A de novo variant of ASXL1 is associated with an atypical phenotype of Bohring-Opitz syndrome: case report and literature review. Front Pediatr 2021;9:678615. 34527642 10.3389/fped.2021.678615 PMC8435705

[15] Capalbo A Fabiani M Caroselli S. Clinical validity and utility of preconception expanded carrier screening for the management of reproductive genetic risk in IVF and general population. Hum Reprod 2021;6:2050–61. 10.1093/humrep/deab087 34021342

[16] Zheng W Yang C Yang S. Obstetric and neonatal outcomes of pregnancies resulting from preimplantation genetic testing: a systematic review and meta-analysis. Hum Reprod Update 2021;27:989–1012. 34473268 10.1093/humupd/dmab027

[17] Sonigo C Mayeur A Sadoun M. What is the threshold of mature oocytes to obtain at least one healthy transferable cleavage-stage embryo after preimplantation genetic testing for fragile X syndrome? Hum Reprod 2021;36:3003–13. 34568938 10.1093/humrep/deab214

[18] Carlston CM O’Donnell-Luria AH Underhill HR. Pathogenic ASXL1 somatic variants in reference databases complicate germline variant interpretation for Bohring-Opitz Syndrome. Hum Mutat 2017;38:517–23. 28229513 10.1002/humu.23203 PMC5487276

[19] Paulson RJ Sauer MV Francis MM Macaso TM Lobo RA. In vitro fertilization in unstimulated cycles: a clinical trial using hCG for timing of follicle aspiration. Obstet Gynecol 1990;76:788–91. 2216225 10.1097/00006250-199011000-00012

[20] Sha T Wang X Cheng W Yan Y. A meta-analysis of pregnancy-related outcomes and complications in women with polycystic ovary syndrome undergoing IVF. Reprod Biomed Online 2019;39:281–93. 31255606 10.1016/j.rbmo.2019.03.203

[21] Foulot H Ranoux C Dubuisson JB Rambaud D Aubriot FX Poirot C. In vitro fertilization without ovarian stimulation: a simplified protocol applied in 80 cycles. Fertil Steril 1989;52:617–21. 2680620 10.1016/s0015-0282(16)60974-3

[22] Berry KA Baron IS Weiss BA Baker R Ahronovich MD Litman FR. In vitro fertilization and late preterm preschoolers’ neuropsychological outcomes: the PETIT study. Am J Obstet Gynecol 2013;209:356.e1–6. 23816840 10.1016/j.ajog.2013.06.041

[23] Lazaraviciute G Kauser M Bhattacharya S Haggarty P Bhattacharya S. A systematic review and meta-analysis of DNA methylation levels and imprinting disorders in children conceived by IVF/ICSI compared with children conceived spontaneously. Hum Reprod Update 2014;20:840–52. 24961233 10.1093/humupd/dmu033

[24] Chen W Peng Y Ma X. Integrated multi-omics reveal epigenomic disturbance of assisted reproductive technologies in human offspring. EBioMedicine 2020;61:103076. 33099088 10.1016/j.ebiom.2020.103076 PMC7585147

[25] Avila M Kirchhoff M Marle N. Delineation of a new chromosome 20q11.2 duplication syndrome including the ASXL1 gene. Am J Med Genet A 2013;161A:1594–8. 23704076 10.1002/ajmg.a.35970

[26] Zhao J Hou Y Fang F. Novel truncating mutations in ASXL1 identified in two boys with Bohring-Opitz syndrome. Eur J Med Genet 2021;64:104155. 33529703 10.1016/j.ejmg.2021.104155

[27] Efthymiou S Salpietro V Pironti E. A de novo truncating mutation in ASXL1 associated with segmental overgrowth. J Genet 2019;98:108. 31819025 10.1007/s12041-019-1155-5 PMC7116628

[28] Katoh M Katoh M. Identification and characterization of ASXL3 gene in silico. Int J Oncol 2004;24:1617–22. 15138607

[29] Moon S Im SK Kim N. Asxl1 exerts an antiproliferative effect on mouse lung maturation via epigenetic repression of the E2f1-Nmyc axis. Cell Death Dis 2018;9:1118. 30389914 10.1038/s41419-018-1171-z PMC6215009

[30] Lee SW Cho YS Na JM. ASXL1 represses retinoic acid receptor-mediated transcription through associating with HP1 and LSD1. J Biol Chem 2010;285:18–29. 19880879 10.1074/jbc.M109.065862 PMC2804164

[31] Park UH Seong MR Kim EJ. Reciprocal regulation of LXRalpha activity by ASXL1 and ASXL2 in lipogenesis. Biochem Biophys Res Commun 2014;443:489–94. 24321552 10.1016/j.bbrc.2013.11.124

